# Composite Resin Preheating Techniques for Cementation of Indirect Restorations

**DOI:** 10.1155/2022/5935668

**Published:** 2022-03-23

**Authors:** Déborah Lousan do Nascimento Poubel, Ana Elisa Ghanem Zanon, Júlio César Franco Almeida, Liliana Vicente Melo de Lucas Rezende, Fernanda Cristina Pimentel Garcia

**Affiliations:** Department of Dentistry, School of Health Sciences, University of Brasilia (UnB), Federal District, DF, Brazil

## Abstract

**Purpose:**

Resin-based materials have been preheated by using different techniques and commercial devices. However, a consensus on the clinical protocol for cementing with preheated composite resins is lacking. The aim of this scoping review was to identify the different methods used for heating composite resins as used for cementing indirect adhesive restorations and to determine the benefits and limitations. *Study Selection*. A search was performed on PubMed/MEDLINE, Embase, Cochrane, Web of Science, Scopus, LIVIVO, and the nonpeer-reviewed literature database. Studies on preheating composite resins for cementing indirect restorations were included, with no restrictions on the type of study, year of publication, or language. The following data were extracted: preheating technique, the device used for preheating, preset temperature, and warming time.

**Results:**

In total, 304 studies were identified. After removing duplicates, 270 articles were selected, and 14 articles were included in the final evaluation. Half of the included studies reported similar preheating techniques using the Calset device for composite resins. The temperatures of 54°C and 68°C were most frequently reported, with a mean warming time of 5 minutes.

**Conclusions:**

Preheating composite resins for the cementation of indirect restorations reduces viscosity, but the material must be used promptly after removal from the device. *Practical Implications*. Different methodologies for preheating composite resins have been reported and used in clinical dental practice. To achieve good results and guide the clinician on use, the techniques for heating composite resins for cementation need to be standardized. Keeping the material warm until the restorative procedure, the thickness of the indirect restoration, and the composition of the composite resins can directly affect the outcome of the procedure.

## 1. Introduction 

Following the concept of adhesion, as described by Buonocore in 1955 [1], the basic concepts of dentistry have changed, with a focus on the most conservative intervention. Among the restorative treatments available, direct composite resin restorations are indicated for teeth with minor loss of structure, while indirect restorations are indicated for teeth with significant functional, structural, and/or esthetic deficiencies [2]. The indirect restorative treatment, which includes laminates, onlays/inlays, and crowns using ceramic, metal, or laboratory-fabricated composite resin as materials, allows for better mechanical properties and marginal adaptation when compared with directly placed composite resins, avoiding polymerization shrinkage and improving wear resistance [3]. Ceramic restorations are resistant to fatigue, with low thermal conductivity and satisfactory biocompatibility [3]. Moreover, indirect restorations which are cemented to the prepared teeth using luting types of cement have better marginal adaptation [4, 5]. Of the various materials available for cementation, resin types of cement, available in light-, chemically, or dual-polymerized forms, are currently preferred because of their hardness, low solubility in oral fluid, and micromechanical bonding to enamel and dentin [2]. Given the different indirect restorative types of cement, other materials, including different types of composite resins, have been studied for this purpose. Light-polymerized composite resins have advantages over dual-polymerized resin types of cement that include stain resistance, color stability, and mechanical wear resistance because of increased inorganic filler loading [6, 7]. The high inorganic filler content directly influences the viscosity of the composite resin, making it less fluid and leading to a thicker, undesirable, cementation line at the adhesive interface [8].

As an alternative to reduced viscosity luting agents, the preheating of composite resins has been suggested [8–15]. By increasing the temperature from 54°C to 70°C, the degree of conversion of the resin becomes similar to that of dual-polymerizing resin cement, the consistency of flow improves, and a thinner cementation line becomes possible [8, 10, 14, 16, 17]. Different preheating techniques, devices, temperatures, heating durations, and transport methods have been reported [8, 11, 13].

A systematic review of the heating and preheating of dental restorative materials (composite resins and glass ionomer types of cement) has recently been published [16]. Although the authors concluded that the preheating technique can improve the physical and mechanical properties of these materials, clinical studies to confirm the advantages of this technique in improving restoration performance are lacking [16]. Many studies have demonstrated the performance of preheating different materials [8, 10, 14, 16], but there remains a lack of evidence that preheating of restorative materials improves the quality and durability of indirect restorations.

Thus, this study aimed to review the influence of the heating protocols for and methods of preheating composite resins used as the luting agent for indirect adhesive restorations.

## 2. Materials and Methods

### 2.1. Selection Criteria and Search Methods

This scoping review was performed according to The Joanna Briggs Institute (JBI) Reviewers Manual 2015-Methodology for JBI Scoping Reviews [18] and Preferred Reporting Items for Systematic reviews and Meta-Analyses extension for Scoping Reviews (PRISMA-ScR) Checklist [19]. It was registered at the Open Science Framework (https://osf.io) under the number DOI: 10.17605/OSF.IO/GXMQE.

The studies were selected according to the eligibility criteria based on the PCC strategy [20] as follows: population (P), composite resins; concept (C), preheating techniques; and context (C), cementation of indirect restorations. There were no restrictions on language, date, or type of studies. The exclusion criteria were studies that investigated resin types of cement, preheating associated with restorative techniques other than cementation of indirect restorations, or studies that analyzed only composite resin properties.

Studies were screened using a search strategy adapted for the following electronic databases: PubMed (MEDLINE), Embase, Cochrane, Scopus, Web of Science, and LIVIVO (Table 1). The search strategy was developed by using the MeSH terms and associated terms. Hand searches were performed on the reference lists to identify additional studies. In addition, non-peer-reviewed studies (OpenGrey, Proquest, and Google Scholar) were searched by screening the titles and abstracts. The first 100 hits were selected (filtered by ‘relevance') on Google Scholar. A specialist opinion was also consulted. Duplicate studies were excluded by using the EndNote Web [21] and Rayyan [22] software programs. The search was conducted on July 21, 2020. A new search was conducted on January 4, 2021, and additional studies were included.

### 2.2. Data Collection

A Kappa test (K) was applied to measure the calibration between the first and second reviewers. After analyzing 10% of all included studies, a 0.81 value of Kappa was obtained. The study selection followed three steps. First, two investigators screened the titles of the studies that appeared to meet the inclusion criteria. In the second phase, the same reviewers independently read the abstracts of potentially relevant articles. Finally, they independently read the full text of the selected articles and excluded those that did not meet the inclusion criteria. Disagreements at any of the three stages were resolved by discussion and mutual agreement among the reviewers. If no consensus was reached, a third author was consulted to reach the final decision.

### 2.3. Data Analyses

Data extraction was performed by the first investigator and followed the mean characteristics of the study: author(s), year of publication, objective, conclusion, primary and secondary testing methods, number of specimens, materials used, preheating device, temperature tested, mean preheating duration, and all details relating to the technique. The second author examined all the retrieved information for the analysis. In an attempt to retrieve missing information, the corresponding author of the studies was contacted when important data were not described in the studies. Information was obtained from two of the four authors contacted.

## 3. Results

### 3.1. Description of Studies

**Figure 1:**
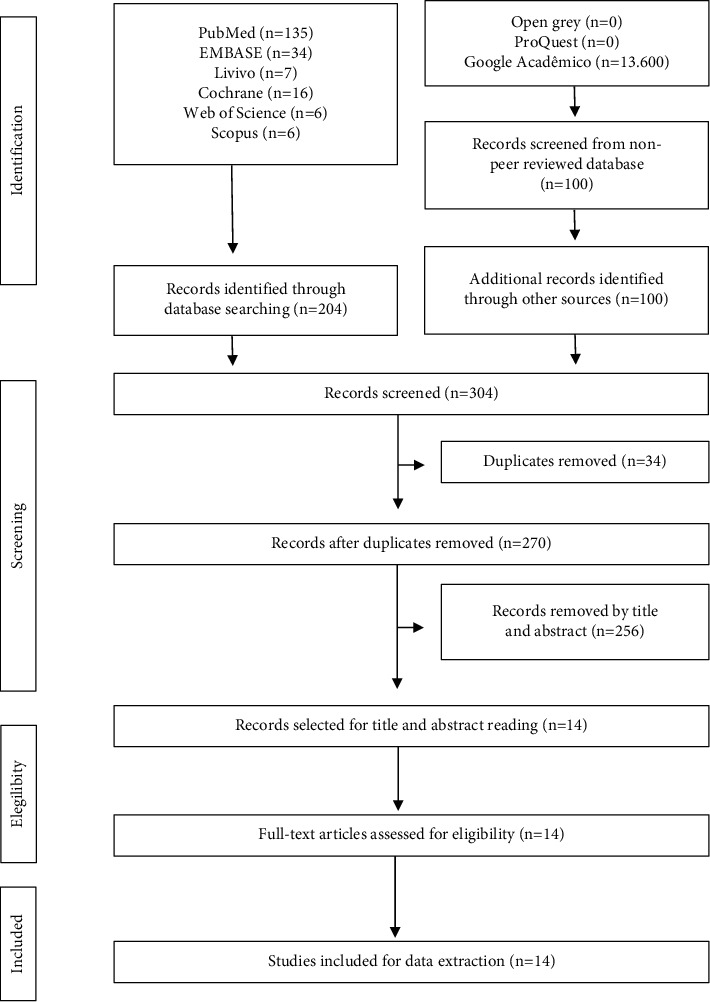
Description of included records in the scoping review.

Based on the search strategy, 304 studies were identified, including the first 100 studies detected on Google Scholar. After the removal of duplicates, 270 studies were analyzed by title and abstract. The inclusion process resulted in 14 studies [11, 13, 23–34] in the second phase. Of these, 12 were in vitro research studies [11, 13, 23–27, 30–34] and two were clinical case reports [28, 29]. The flow chart was adapted from PRISMA [20] and is illustrated in Figure 1. The timeline of the publications in the English language and from six countries, namely, Brazil [13, 27–30], Chile [28], the United States [23, 24, 26, 31–34], Italy [11], the Czech Republic [25], and Thailand [29] from 2009 to 2018 is shown in Table 2 [35].

### 3.2. Preheating Device

Ten studies used the Calset oven (AdDent Inc.) device to preheat composite resin [11, 23, 24, 26, 29–34]. The other preheating devices described were the Digital wax pot (SJK) [13], ENA heat (Micerium S.p.A) [25], and Wax Heater Pot 4 (manufacturer not mentioned) [28]. Only one study used an incubator (manufacturer not mentioned) for preheating, but its specifications were not provided [27].

### 3.3. Temperatures and Preheating times

The temperatures used to preheat composite resins were 54°C [11, 23, 29, 32, 34], 55°C [25], 58°C [28], 60°C [27], 64°C [13], and 68°C [24, 30, 33]. The reported range was from 54°C to 68°C. The temperatures of 54°C and 68°C were most frequently reported. Two studies did not report the preheating temperature [26, 31]. A warming time of 5 min was specified in eight studies [13, 23, 24, 26, 28, 31, 32, 34] and of 30, 15, and 60 min in three other studies [25, 27, 29]. Three studies did not report the time used for heating composite resins [11, 30, 33].

### 3.4. Preheating Methods and Mean Required Time of the Clinical Procedure

Only one study reported details related to the glass container in which the resin was placed when heated in the device [27]. Almeida et al. [27] reported removing the increment from the oven and immediately applying it to the ceramic. However, the transport time was not specified. Goulart et al. [13] stated that the time taken for the material to be removed from the heating device until the assessed property was measured was less than 30 s. Other studies did not report this aspect.

### 3.5. Composite Resins Tested and Light-Polymerizing Units

Seven studies preheated the microhybrid composite resin Filtek Z100 (3M ESPE) [23, 24, 26, 30, 32–34]. Other microhybrid composite resins used were Gradia Direct posterior (GC) [31] and Venus1 (Kulzer) [11, 13]. Four articles preheated composite resins with nanohybrid particles, including Filtek Z350 XT (3M ESPE) [27], Tetric N-Ceram (Ivoclar-Vivadent) [28], Z250 XT (3M ESPE) [13], Miris 2 (Coltene-Whaledent) [29], and other composite resins such as Enamel Plus Hri (Micerium) [25]. Goulart et al. [13] compared the use of composite resins at room temperature (±23°C) for cementing indirect restorations. Acquaviva et al. [11] conducted a study comparing composite resin at room temperature, heated composite resin, and resin cement. Another five studies focused on the preheating of composite resin and resin cement [13, 24, 27] for cementing indirect restorations. Two clinical case reports [28, 29] and seven in vitro studies [23, 24, 26, 30–34] used heated composite resin. In no study were details provided regarding the amount of preheated composite resins used for cementing indirect restorations.

The following brands of the light-polymerization unit were used: halogen lamp Swiss Master Light1 (EMS, Neun, Switzerland), FlashLight (Dental Discus), Optilight Max (Gnatus), Den-Mat (Allegro), and Valo (Ultradent). The light-polymerization power was between 400 mW/cm^2^ and 1200 mW/cm^2^, and the duration ranged from 40 s to 120 s.

### 3.6. Property Testing

From the included in vitro studies, different properties of composite resins were evaluated, including the degree of conversion [11], color stability [27], microtensile bond strength-adhesive interfaces [13], fatigue resistance [23, 26, 30–34], vertical seating [24], and vertical marginal discrepancy [25].

## 4. Discussion

Indirect adhesive restorations can be cemented with preheated composite resins yielding reduced viscosity materials that provide a clinically acceptable cement film thickness and have better mechanical properties than those of conventional types of cement [8–11, 14]. Despite clinical and laboratory evidence suggesting the advantages of preheating composite resins [8–11, 14], their protocol for use as a cementation agent for indirect restorations has not yet been fully elucidated. Advantages reported in studies on preheating resin materials include an increased degree of conversion [10], improved marginal adaptation of restorations because of reduced viscosity [36], and decreased polymerization contraction [37]. However, the methodologies used in the preheating of composite resins, their mechanical properties, and their performance as luting types of cement for indirect restorations must be analyzed.

The composite resin is typically preheated in a device that is programmed to reach a certain temperature that should be confirmed for accuracy and monitored and controlled during storage in the heater [38]. The temperature of the preheated composite resin cools rapidly when removed from the heating device, approximately 50% in 2 min [38]. Thus, the material should be placed, adapted, the restoration seated, and light-polymerized rapidly. When the high temperature is maintained, monomer conversion will be greater than at room temperature (±23°C) [11].

Seven of the included articles reported similar preheating techniques with a commercially available device (Calset, AdDent Inc.) that the manufacturer claims preheats and stores composite resins at temperatures of 54°C, 60°C, or 64°C until they are ready for use. Composite resin syringes can be heated and the resin can then be directly injected onto the restoration or prepared toot, reducing the clinical time [8, 38, 39]. Despite what was specified by the manufacturer, Daronch et al. [38], who used Calset (Addent Inc.) in their study, reported that the maximum temperature reached was 48.3°C and 54.7°C when the preset temperature of the device was set at 54°C and 60°C, respectively. The equipment (ENA heat, Micerium) used for heating composite resins in the study by Mounajjed et al. [25] was preset at temperatures from 39°C to 55°C. A temperature of 55°C was recommended by the manufacturer for heating composite resins for cementation and had six spaces for heating syringes of composite resin.

Previous studies evaluated the use of this heating equipment [40–42], but Goulart et al. [13] and Olivares et al. [28] used wax pot heaters in their studies (Digital wax pot, SJK and Wax Heater Pot 4, Denshine) because these devices allowed setting the temperature according to clinical needs. The preheating of composite resins in a wax pot heater has also been described in another study where the heating time of the composite resins was around 2 to 3 min [11]. The authors concluded that the wax pot was a straightforward, rapid, and economical option [43]. Almeida et al. [27] used an incubator oven to heat composite resins at 60°C for 30 min. However, they did not provide manufacturer specifications for the equipment. However, a preheating technique in a bacteriological oven (model 502, Fanem) at a temperature of 54°C has been described [44].

Composite resins can be heated in different dry heating devices, as long as the temperature is controlled and remains stable until clinical use. The use of a specifically marketed device such as Calset (AdDent Inc.) facilitates the technique and ensures standardization of the preheating process. The use of parallel heaters requires the preset temperature of the device to be measured and checked until the required temperature is reached.

The temperatures for preheating composite resins described in the studies ranged from 54°C to 68°C, and this range has been considered ideal for improving the working properties of the material [11, 13, 23–27, 30–34]. Daronch et al. [10] evaluated the degree of conversion at temperatures ranging from 3°C to 60°C and reported that, at the highest temperature, a greater degree of conversion was reached. Daronch et al. [38] used the Calset device (AdDent Inc.) and reported a drop in the degree of conversion after a certain temperature because of degradation of the photoinitiator. In monomers such as bisphenol A diglycidyl ether dimethacrylate (Bis-GMA) or ethoxylated bisphenol A dimethacrylate (Bis-EMA), the volatility limit of dimethacrylate monomers used in resin formulations occurs close to 90°C, a temperature that could damage some composite resin components and harm pulpal tissue. However, 90°C is above the maximum temperature allowed by the heating device. In addition, because of incomplete polymerization, unreacted monomers may leach into the saliva, promoting undesirable consequences, and the loss of plasticizers may decrease mechanical strength, dimensional stability, and color change and allow bacterial growth. Unreacted monomers can also cause allergic and sensitivity reactions [45].

The heating time for the composite resin inside the heater is also an important evaluation parameter, with the average time for the device to reach both tested preset temperatures (54°C or 60°C) being 11 min [38]. Therefore, in addition to ensuring that the heating device can maintain a controlled and predefined temperature, the temperature should be reached in a predictable time. A drop in the temperature of the composite resin was reported between its removal from the heating device and the mouth, estimated to be 50% after 2 min and 90% after 5 min when heated to 60°C and removed from the device, indicating the need for calibration during all processes. In addition, heated composite resins have been reported to provide better results than composite resins at room temperature [10, 16, 38]. Composite resins with different compositions can take different times to reach a stable temperature, and some low-molecular-weight components of the photoinitiator system can be volatilized with prolonged heating [10, 38]. Therefore, different heating methods have been used for in vitro studies and for clinical techniques. Lopes et al. [16] reported that some studies used a reasonable clinical time of approximately 15 min.

The temperature must be controlled to avoid causing pulpal damage, but increasing the composite resin temperature to between 54°C and 60°C does not significantly change the intrapulpal temperature [46]. Lopes et al. [16] noted that dentin thickness acts as a thermal barrier, preventing harmful stimuli and protecting the dental pulp.

According to manufacturers, the Calset (AdDent Inc.) and ENA heat (Micerium) devices are designed to attach a syringe, acting as a container. The wax pot heaters (Digital wax pot, SJK and Wax Heater Pot 4, Denshine) can directly heat the composite resin inside of the syringe. Comparing the two preheating methods, Daronch et al. [38] concluded that the composite resins already assembled in the application syringe showed a significantly higher maximum temperature (36.6°C ± 2.2°C) than the composite resins that were heated without a container (33.6°C ± 0.5°C). Thus, the composite compule preloaded into a delivery syringe was more efficient. Higher temperatures were attained with this method as opposed to preheating the compule separately [16].

The way the composite resins are arranged and placed in the preheating device affects its clinical application, as the working time should be minimum owing to the decrease in temperature after its removal from the heater [10, 38]. Once the composite resin is attached to a syringe or loaded into the indirect restoration to be cemented, the dentist can simply remove and apply it to the teeth, without concerns about assembling the application system, thus reducing the working time, and maintaining the temperature as high as possible [38].

The viscosity of composite resins is linked to factors in their composition such as the organic matrix and amount and size of inorganic fillers [47]. Analyzing the particle size, materials with smaller particles appear more fluid when compared with those that contain bigger particles. Regarding the amount of filler, the higher the filler load, the higher the viscosity [46]. The amount and type of monomer can also cause an increase in viscosity, as monomers such as Bis-GMA and urethane dimethacrylate are quite viscous, whereas Bis-EMA and triethylene glycol dimethacrylate are more fluid [47, 48]. Among the studies included, Goulart et al. [13] compared the use of two preheated composite resins, a microhybrid (Venus 1, Kulzer) and a nanohybrid (Z250 XT, 3M ESPE) with the same preheating protocol and mechanical test. The nanohybrid resin, as evaluated by SEM after cementation, formed a thicker film than the microhybrid resin.

The degree of conversion can be increased by preheating, decreasing the light-polymerization time, and maintaining a degree of conversion similar to or even better than when the composite resins are irradiated for longer at 22°C [10, 11]. Preheating the resins to 60°C increased the conversion of monomers by increasing molecular mobility. Compounds with higher conversion have greater crosslinking, reducing the free space of the polymers and improving their mechanical properties [10]. A 5 s light-polymerization time with a composite resin preheated to 57°C resulted in a higher conversion rate than that observed after a 40 s exposure at 22°C [10]. According to Acquaviva et al. [11], the thickness of the onlays affected the degree of conversion of both composite resins and dual-polymerizing types of cement, and an excellent degree of conversion can be achieved by preheating the light-polymerizing composite resins. No ideal light-polymerizing time or intensity has yet been determined. Thus, purely light-polymerizing types of cement or resins must be used with care, as there must be enough light to pass through the materials for adequate conversion of the monomers. If an indirect restoration is thinner than 2 mm, the light passage should be adequate [49].

A consensus on the limits for a clinically acceptable film thickness is lacking. Marcondes et al. [50] stated that composite resins, being restorative materials, are designed to provide intraoral resistance. Therefore, an increased cementation thickness, even if it exceeds the value defined by ISO 4049 [51], should be clinically acceptable. Composite resins are designed for color stability and abrasion resistance, as shown in laboratory and clinical studies [10, 11, 38, 49, 50]. In general, when composite resins are preheated, viscosity is reduced and adaptation to cavity walls is improved [50].

The presence of amines in chemically polymerized resins, including dual-resin types of cement, may eventually result in the staining of indirect restorations, and, therefore, they should be avoided for cementation of translucent or thin restorations [7]. Almeida et al. [27] reported that dual-resin types of cement showed greater color variation than preheated composite resins, light-polymerized resin types of cement, and flowable composite resins. Goulart et al. [13] reported that preheating composite resins did not increase the cementation bond strength of indirect restorations, even though increased mechanical properties have been reported because of increased conversion [8, 12]. The results could be explained by the loss of temperature of the material during the bonding procedure and not reaching an adequate degree of conversion. In contrast, the conclusion was that the material can still be used to reduce its viscosity and improve the fit of the restoration [13]. The use of different luting agents provides many alternatives for cementation, which also can produce varying results in a vertical discrepancy of the definitive restoration [52]. Mounajjed et al. [25] compared the vertical marginal discrepancy of pressed crowns of lithium disilicate by using different cementing agents. The preheated composite resin Enamel Plus HRi (Micerium) obtained higher values of marginal discrepancies than a cement flow resin (Harvard Premium Flow) and the dual-resin cement, RelyX Ultimate (3M ESPE). The authors stated that the methodology used may have affected the results of the study, for example, using a specimen at low temperature, which could have reduced the fluidity of the resin, and the difficulty of standardizing the seating pressure at different viscosities. Magne et al. [24] investigated the vertical displacement of composite resin inlays, onlays, and computer-aided design and computer-aided manufactured overlays. The preheated composite resin used in cementation resulted in the better seating of inlays, onlays, and overlays than the dual-polymerizing resin cement [24].

## 5. Conclusion

This scoping review observed a large variation in the use of preheating techniques on composite resins used for cementation of indirect restorations. No consensus was found regarding the recommended preheating devices, heating durations, or temperatures for this clinical procedure. Some aspects can be considered relevant when considering preheating techniques for composite resins. (1) All heating devices demonstrated effectiveness in heating composite resins used for the cementation of indirect restorations. (2) The ideal heating device must be free of moisture and calibrated to reach a predetermined temperature (between 54°C and 68°C) on heating and must maintain stability at the predetermined temperature after heating. (3) The preheated material must be used as soon as possible after being removed from the device, as the temperature of the composite resin will decrease quickly. (4) Preheating the material directly in the prosthetic restoration or the dispensing syringe reduces clinical time. (5) The indirect restoration must be less than 2 mm thick if a light-polymerizing luting cement or composite reins is to be used. (6) The composition of the composite resins directly affects the viscosity reached after preheating. Therefore, materials indicated for this purpose or that show increased fluidity when heated should be used.

Based on the results of the included studies, more research is needed on preheating techniques for composite resins used for the cementation of indirect restorations; in particular, longitudinal clinical evaluations are needed. Furthermore, studies correlating the composition of composite resins and their behavior when heated are required.

## Figures and Tables

**Table 1:** Electronic database and search strategy (PubMed).


((“Composite resins”[MeSH terms] OR “composite resin”[All fields] OR “composite dental resin”[MeSH terms] OR “composite dental resin”[All fields] OR “composite dental resin”[All fields] OR “bisphenol a-glycidyl methacrylate”[MeSH terms] OR “bisphenol a-glycidyl methacrylate”[All fields] OR “composite properties”[All fields] OR “composite dental material”[All fields] OR “composite dental restorative”[All fields] OR “composite dental restoratives”[All fields] OR “composite dental restorative material”[All fields] OR “composite dental restorative materials”[All fields] OR “composite dental filling”[All fields] OR “composite dental filling material”[All fields] OR “composite dental filling materials”[All fields] OR “methacrylate, bisphenol A-Glydidyl”[All fields] OR “Bis(Phenol A-Glycydyl Methacrylate)”[All fields] OR “Bis-GMA”[All fields] OR “bis-GMA”[All fields] OR “bisphenol A-Glycidyl methacrylate Homopolymer”[All fields] OR “bisphenol a-glycidyl methacrylate Homopolymer”[All fields] OR “Bis(Phenol A-Glycidyl methacrylate), Homopolymer”[All fields] OR “Poly(Bis-GMA)” [all fields] OR “Bis-GMA Resin”[All fields] OR “bis-GMA Resin”[All fields] OR “Bis-GMA Resins”[All fields] OR “resin, Bis-GMA”[All fields] OR “resins, Bis-GMA”[All fields] OR “bisphenol A-Glycidyl methacrylate Polymer”[All fields] OR “bisphenol a glycidyl methacrylate Polymer”[All fields] OR “2-propenoic acid, 2-methyl-, (1-methylethylidene)bis(4,1-phenyleneoxy(2-hydroxy-3,1-propanediyl)) ester, homopolymer”[All fields] OR “Bis-GMA Polymer”[All fields] OR “bis-GMA Polymer”[All fields] OR “Bis-GMA Polymers”[All fields] OR “polymer, Bis-GMA”[All fields] OR “polymers, Bis-GMA”[All fields]) AND (“preheat”[All fields] OR “preheated”[All fields] OR “preheating”[All fields] OR “hot temperature”[MeSH terms] OR “hot temperature”[All fields]) AND (“cementation”[MeSH terms] OR “cementations”[MeSH terms] OR “cementation”[All fields] OR “cementations”[All fields] OR “dental cement”[All fields] OR “dental cements”[MeSH terms] OR “dental cements”[All fields] OR “luting agent”[All fields] OR “luting agents”[All fields] OR “cementation agents”[All fields] OR “cementation agent”[All fields] OR “cement, Dental”[All fields] AND “permanent dental restoration”[MeSH terms] OR “permanent dental restorations”[All fields] OR “restorations, permanent Dental”[All fields] OR “dental restoration, Permanent”[All fields] OR “restoration, permanent Dental”[All fields] OR “dental restorations, Permanent”[All fields] OR “dental permanent Fillings”[All fields] OR “filling, permanent Dental”[All fields] OR “permanent dental Fillings”[All fields] OR “permanent fillings, Dental”[All fields] OR “permanent filling, Dental”[All fields] OR “dental filling, Permanent”[All fields] OR “dental permanent Filling”[All fields] OR “filling, dental Permanent”[All fields] OR “filling, permanent Dental”[All fields] OR “permanent dental Filling”[All fields] OR “fillings, dental Permanent”[All fields] OR “dental fillings, Permanent”[All fields]))

**Table 2:** Main characteristics of the included studies.

*N*	Author, year, country	Total “*n*” of specimens	Heated composite resin trademark, classification, color, and volume	Preheating device	Temperature, warm-up time, means of transport, and transport time	Valued property and assessment device	Light curing trademark, light curing time, and characteristics	Valued property control group (non-preheated)
1	Acquaviva et al., 2009, Italy [11]	180; 5 preheated	Venus1 (kulzer); microhybrid composite resin; N/A; N/A	Calset (AdDent Inc.)	54°C; N/A; N/A; N/A	Degree of conversion; spectrometer micro-Raman dilor (HR LabRam)	Halogen lamp swiss master Light1 (EMS); 40 s, 60 s, 120 s; 1200 mW/cm^2^; 800 mW/cm^2^; 400 mW/cm^2^	Calibra1 (dentsply), dual-cured resin cement; Variolink1 II (ivoclar-vivadent), dual-cured resin cement; Venus1 (kulzer), microhybrid composite resin
2	Almeida et al., 2015, Brazil [27]	40; 10 preheated	Filtek Z350 XT (3 M/Espe); nanohybrid composite resin; A1; N/A	Incubator (N/A)	60°C; 30 min; glass container; “immediately”	Color stability; spectrophotometer (easyshade, vita zahnfabrik)	FlashLight (discus dental); 1 min; 800 mW/cm^2^	RelyX ARC (3 M/Espe), dual-cured resin cement; RelyX veneer (3 M/Espe), light-polymerizing cement; Filtek Z350 flow (3 M/Espe), flowable
3	Goulart et al., 2018, Brazil [13]	50; 50 preheated	Venus1 (kulzer); microhybrid composite resin; A2; N/A and Z250 XT (3 M/Espe); nanohybrid	Digital wax pot (SJK)	64°C; 5 min; N/A; “reduced to 30 s”	Microtensile bond strength and adhesive interfaces; stereomicroscope (EMZ, Meji Techno)	Optilight max (gnatus); 40 s; 900 mW/cm^2^	Venus1 (Kulzer), microhybrid composite resin color A2; Z250 XT (3M), microhybrid composite resin color A2; RelyX ARC (3M), dual-cured resin
4	Magne et al., 2009, United States [32]	30; 30 preheated	Filtek Z100 (3 M/Espe); microhybrid composite resin; N/A; N/A	Calset (AdDent Inc.)	54°C; 5 min; N/A; N/A	Fatigue resistance; closed-loop servohydraulics (Mini Bionix II, MTS Systems)	N/A; 60 s; N/A	None
5	Magne et al., 2009, United States [34]	30; 30 preheated	Filtek Z100 (3 M/Espe); microhybrid composite resin; N/A; N/A	Calset (AdDent Inc.)	54°C; 5 min; N/A; N/A	Fatigue resistance; closed-loop servohydraulics (Mini Bionix II, MTS Systems)	Allegro (den-mat); 60 s; N/A	None
6	Magne et al., 2010, United States [33]	30; 30 preheated	Filtek Z100 (3 M/Espe); microhybrid composite resin; N/A; N/A	Calset (AdDent Inc.)	68°C; N/A; N/A; N/A	Fatigue resistance; closed-loop servohydraulics (mini bionix II, MTS systems)	Allegro (den-mat); 60 s; 1000 mW/cm^2^	None
7	Magne et al., 2011, United States [23]	28; 28 preheated	Filtek Z100 (3 M/Espe); microhybrid composite resin; N/A; N/A	Calset (AdDent Inc.)	54°C; 5 min; N/A; N/A	Fatigue resistance; closed-loop servohydraulics (Mini Bionix II, MTS Systems)	Valo (ultradent); 60 s; 1000 mW/cm^2^	None
8	Magne et al., 2018, United States [24]	60; 30 preheated	Filtek Z100 (3 M/Espe); microhybrid composite resin; N/A; N/A	Calset (AdDent Inc.)	68°C; 5 min; N/A; N/A	Vertical seating; acumen III (MTS systems)	Valo (ultradent); 60 s; N/A	RelyX ultimate cement (3M), dual-cured resin cement
9	Mounajjed et al., 2017, Czech Republic [25]	18; 6 preheated	Enamel plus HRi (Micerium S.p.A); nanohybrid composite resin; N/A; N/A	Heater ENA heat (micerium S.p.A)	55°C; 1 hour; N/A; N/A	Vertical marginal discrepancy; microscopy at x200 magnification with special image analysis software (Keyence)	Valo (ultradent); 60 s; N/A	Harvard PremiumFlow cement (GmbH), nanohybrid composite resin; RelyX ultimate cement (3 M/Espe), dual-cured resin cement
10	Oderich et al., 2011, United States [26]	60; 60 preheated	Filtek Z100 (3 M/Espe); microhybrid composite resin; N/A; N/A	Calset (AdDent Inc.)	N/A; 5 min; N/A; N/A	Fatigue resistance; closed-loop servohydraulics (Mini Bionix II, MTS Systems)	Valo (ultradent); 60 s; N/A	None
11	Olivares et al., 2011, Chile [28]	10; 10 preheated	Tetric N-ceram (Ivoclar-vivadent AG); nanohybrid composite resin; A2; N/A	Wax Heater Pot 4 (Denshine)	58°C; 5 min; N/A; N/A	None	N/A; 60 s; N/A	None
12	Rickman et al., 2011, Thailand [29]	7; 7 preheated	Miris 2 (coltene-whaledent); nanohybrid composite resin; A2; N/A	Calset (AdDent Inc.)	54°C; 15 min; N/A; N/A	None	N/A; N/A; N/A	None
13	Schlichting et al., 2011, Brazil [30]	40; 40 preheated	Filtek Z100 (3 M/Espe); microhybrid composite resin; N/A; N/A	Calset (AdDent Inc.)	68°C; N/A; N/A; N/A	Fatigue resistance; closed-loop servohydraulics (Mini Bionix II, MTS Systems)	Allegro (den-mat); 60 s; 1000 mW/cm^2^	None
14	Soares et al., 2018, United States [31]	45; 30 preheated	Gradia direct posterior (GC); microhybrid composite resin; N/A; N/A	Calset (AdDent Inc.)	N/A; 5 min; N/A; N/A	Fatigue resistance; closed-loop servohydraulics (Mini Bionix II, MTS Systems)	Valo (ultradent); 60 s; 1000 mW/cm^2^	None

Table 2 is reproduced from “Técnicas de aquecimento de resinas compostas para cimentação de restaurações indiretas: Scoping review” © 2022 by Zanon AEG, Poubel DLN, and Garcia FCP under CC BY 4.0 (http://creativecommons.org/licenses/by/4.0/) [35]. N/A: not available; none: not applicable.

## Data Availability

The data used to support the study are available from the corresponding author upon request.
